# Panniculite mésentérique associée à une pancréatite aiguë: à propos d’une observation

**DOI:** 10.11604/pamj.2016.24.206.9322

**Published:** 2016-07-08

**Authors:** Hedfi Mohamed, Charfi Mehdi, Messaoudi Ikram, Moussa Myriam, Bouhawala Habib, Chouchene Adnene

**Affiliations:** 1 Service de Chirurgie Générale Hôpital Des FSI La Marsa, Tunisie

**Keywords:** Panniculite mésentérique, pancréatite aigue, perte de poids, Mesenteric panniculitis, acute pancreatitis, loss of weight

## Abstract

La panniculite mésentérique est une inflammation non spécifique du mésentère. Sa présentation clinique est variable en fonction du stade de la maladie Les signes cliniques sont principalement des douleurs mais la moitié des patients restent asymptomatiques. Une masse abdominale palpable, perte de poids nausées et vomissements. Il peut exister un syndrome inflammatoire d’intensité variable. La panniculite est évoquée devant une hyperdensité du mésentère au scanner avec des adhérences avec les organes de voisinage. Histologiquement il existe dégénérescence des adipocytes à l’origine d’une réaction à corps étranger, puis apparaissent des lésions inflammatoires lymphoplasmocytaires. Nous rapportons une nouvelle observation de panniculite mésentérique associé à une pancréatite aiguë.

## Abstract (en)

Mesenteric panniculitis is a nonspecific inflammation involving the mesentery. Its clinical presentation is variable depending on the stage of the disease. Clinical signs are usually pain but half of the patients remain asymptomatic. Palpable abdominal mass, weight loss, nausea and vomiting. There may be an inflammatory syndrome of variable intensity. Panniculitis is suspected based on scan showing hyperdense mesentery with adhesions to adjacent organs. Histologically there is adipocyte degeneration causing foreign body reaction and appear lymphoplasmacytic inflammatory lesions. We report a new case of mesenteric panniculitis associated with acute pancreatitis.

## Introduction

La panniculite mésentérique (PM) est une affection rare qui se définie par une inflammation aiguë ou sub-aiguë du mésentère pouvant associer une fibrose et une rétraction péritonéale lorsqu’elle passe à la chronicité et elle est alors généralement dénommée mésentérite rétractile (MR) [1]. Cette affection est de cause inconnue. La PM débute par une dégénérescence des adipocytes à l’origine d’une réaction à corps étranger, puis apparaissent des lésions inflammatoires lymphoplasmocytaires associées à la présence de macrophages et de quelques éosinophiles. Enfin, survient la prolifération localisée ou diffuse d’un tissu collagène dense avec rétraction du mésentère, accolement et sténose d’anses grêles. Un blocage des voies lymphatiques peut conduire à la formation de pseudo-kystes, à la malabsorption des graisses et à une entéropathie exsudative [1, 2]. Nous rapportons un nouveau cas de panniculite mésentérique associé à une pancréatite aiguë diagnostiqué sur les données du scanner et nous nous proposons d’étudier les particularités diagnostiques et évolutives de cette affection rare.

## Patient et observation

**Figure 1:**
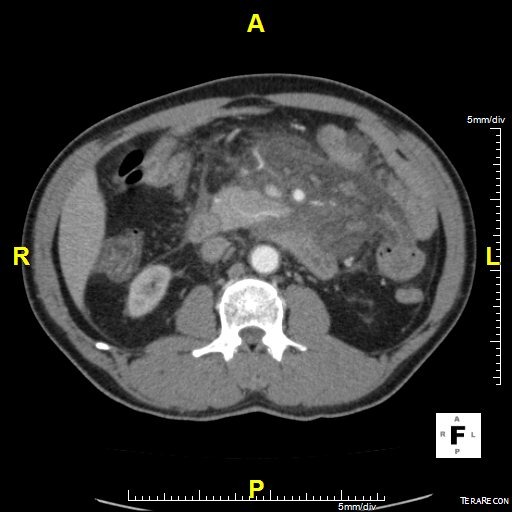
Scanner abdominal coupe transversal pancréas tuméfié avec infiltration mésentérique

**Figure 2:**
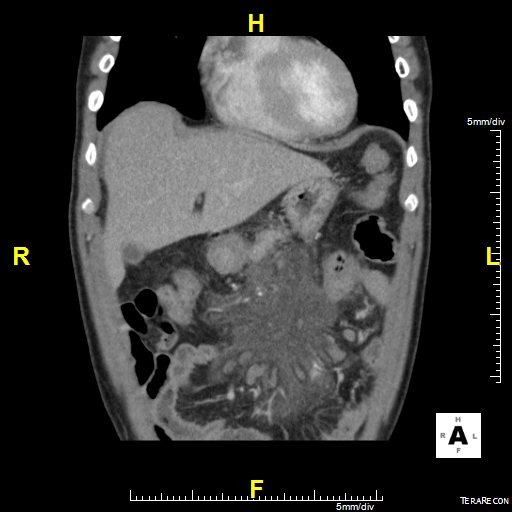
Scanner abdominal coupe longitudinale densification mésentérique et infiltration de la racine du mésentère

**Figure 3:**
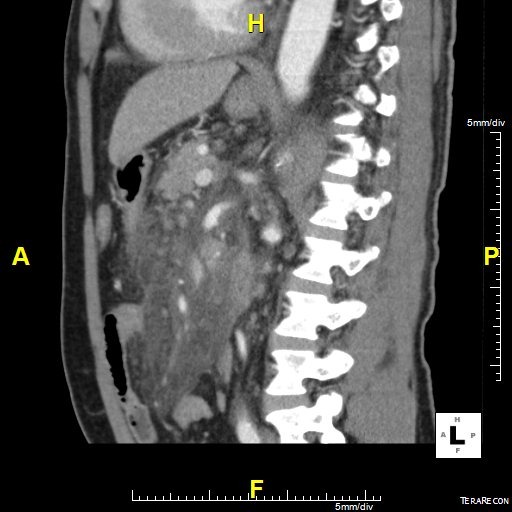
Coupe longitudinale épaississement du mésentère avec engrainement des vaisseaux

**Figure 4:**
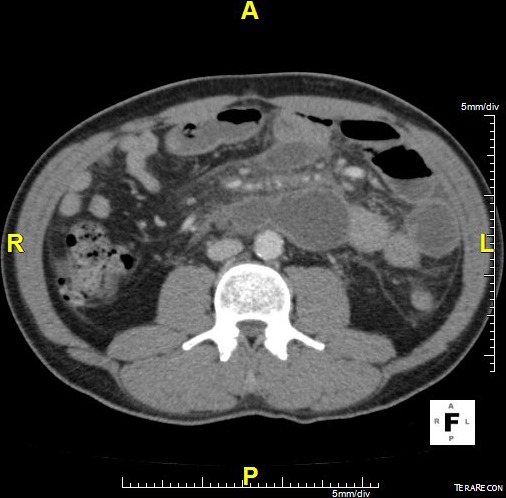
Scanner de contrôle coupe transversale organisation kystique de la panniculite mésentérique

**Figure 5:**
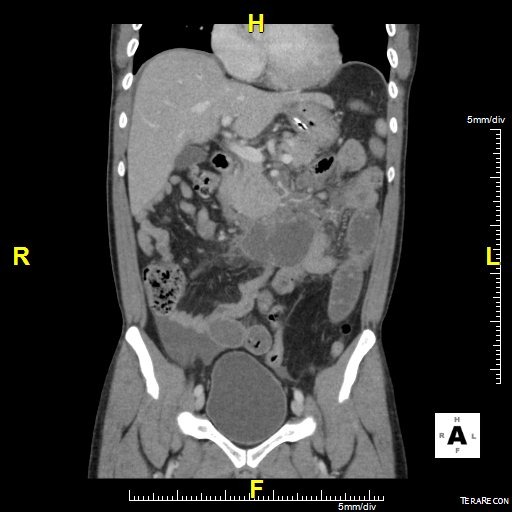
Scanner de contrôle coupe longitudinal organisation kystique de la panniculite mésentérique

Il s’agit d’un patient ag é de 62 ans hypertendu, diabétique et insuffisant rénal au stade d’hémodialyse hospitalisé en urgence pour douleur abdominale épigastrique intense avec vomissement d’apparition récente après un repas copieux. L’examen clinique avait trouvé un patient subfébrile à 37.8°, avec un état hémodynamique stable; l’examen abdominal avait montré une nette sensibilité diffuse avec un maximum au niveau de l’étage sus ombilical. Le reste de l’examen était sans anomalies. La pancréatite aiguë a été suspecté et le bilan biologique avait montré une amylasémie a 4 fois la normale, une lipasémie a 3 fois la normale, le bilan hépatique était normale par ailleurs il y avait une hyperleucocytose a 16200 éléments /mm^3^ et une créatininemie a 165. L’échographie réalisé en urgence avait trouvé une vésicule lithiasique sans signes de cholécystite. Le scanner abdominal fait a 48 heure de l’hospitalisation avait montré un pancréas augmenté de volume sans nécrose intra pancréatique mais avec une infiltration mésentérique dense avec épaississement du mésentère et une infiltration de la graisse et une congestion des vaisseaux; et qui correspond a une panniculite mésentérique plus que de la nécrose pancréatique (Figure 1, Figure 2, Figure 3). L’évolution était favorable sous traitement symptomatique (antalgique et antinflammatoire) ce qui avait permis la sortie du patient; mais 21 jours après sa sortie le patient avait consulté pour reprise de douleurs avec vomissement; le scanner de contrôle trouve une organisation kystique de la panniculite avec compression duodénale (Figure 4, Figure 5). Le traitement médicale par corticoïde IV et aspiration nasogastrique avait permis une amélioration clinique avec régression de l’inflammation mésentérique.

## Discussion

La panniculite mésentérique (PM) constitue un processus inflammatoire rare et chronique du tissue adipeux du mésentère, premièrement décrite par Juraz en 1942. La maladie touche surtout des individus de la cinquantaine, avec un sexe ratio de 2 hommes pour 1 femme [1, 3]. Elle est caractérisée par l´association variable de lésions d´inflammation, de nécrose et de sclérose du tissu adipeux mésentérique. Sa physiopathologie reste mal connue bien que des associations avec des pathologies inflammatoires et carcinomateuses, en particulier lymphomateuses, aient été décrites. Cette maladie peut rester asymptomatique dans 30 à 50 % des cas. Si la maladie est symptomatique, on peut observer généralement des douleurs abdominales (70%), une masse abdominale (40 à 70%) de siège variable, sensible à la palpation (33%). Une asthénie et une fébricule sont quasi-constantes; au stade de Mésentérite Rétractile (MR), on observe généralement une constipation et des épisodes occlusifs. L’état général est altéré. Une masse abdominale peut être observée [1–5]. La tomodensitométrie abdominale est souvent le premier examen d´imagerie permettant d´évoquer le diagnostic et d´écarter les diagnostics différentiels. La tomodensitométrie montre une hyper-atténuation du tissu adipeux du mésentère qui est un signe caractéristique du PM. La densité graisseuse du mésentère (-40 a -60 HU) est supérieure a celle de la graisse souscutanée ou retro péritonéal normal (-100 a -160 HU) [6]. Plus souvent, l’infiltration s’étend au jéjunum et spécialement a la racine du mésentère. Il pousse les anses grêles avoisinantes et enveloppe les vaisseaux mésentériques. L’hyper-atténuation du tissu adipeux du mésentère au PM doit être différenciée d’autres conditions comme le lymphome, tumeurs primaires ou secondaires du mésentère, conditions inflammatoires, trauma, ascites, œdème et hémorragie péritoine [6–8]. Cet aspect tomodensitométrique, de même que l’aspect échographique par IRM, n’étant pas spécifique, une laparotomie exploratrice avec plusieurs biopsies est parfois nécessaire [6]. L’évolution de la PM se fait par poussées, avec dans plus de 50% des cas, une guérison spontanée. Une association avec un lymphome a été rapportée. Dans les autres cas, elle se fait vers la MR avec ses complications, occlusion, thrombose de la veine mésentérique supérieure, anasarque et/ou cachexie. Les corticoïdes pour la poussée et l’azathioprine en entretien sont parfois efficaces. Un petit essai récent a montré une bonne efficacité de la thalidomide au stade de PM [1, 2, 4, 8]. Les lésions de panniculite étant le plus souvent non résécables, le traitement chirurgical est réservé au traitement des complications obstructives digestives ou vasculaires et consiste généralement en la réalisation d´un geste de dérivation ou de résection intestinale segmentaire. L´évolution de la panniculite mésentérique est le plus souvent spontanément favorable avec une résolution complète de la symptomatologie dans un délai variable [1–3, 5, 7, 8].

## Conclusion

La panniculite mésentérique, également appelée mésentérite rétractile est une maladie rare, entraînant un épaississement et un raccourcissement du mésentère. Elle est caractérisée par l'association variable de lésions d'inflammation, de nécrose et de sclérose du tissu adipeux mésentérique. La tomodensitométrie est l’examen du choix pour apporter le diagnostic du panniculite mésentérique, évitant ainsi des biopsies ou laparotomies pas nécessaires. Le Diagnostic précoce peut être bénéfique aux cas d’association avec un néoplasme dans ce cadre nous avons rapporté une nouvelle observation insolite du fait de son association avec une pancréatite aiguë et de son évolution vers l’organisation kystique.
